# Impact of the therapeutic positioning report in the P&R process in Spain: analysis of orphan drugs approved by the European Commission and reimbursed in Spain from 2003 to 2019

**DOI:** 10.1186/s13023-020-01507-4

**Published:** 2020-08-28

**Authors:** Xavier Badia, Tania Vico, John Shepherd, Alicia Gil, José Luis Poveda-Andrés, César Hernández

**Affiliations:** 1 Omakase Consulting S.L., Barcelona, Spain; 2 Hospital Universitario y Politécnico de La Fe, Valencia, Spain; 3 Agencia Española de Medicamentos y Productos Sanitarios, Madrid, Spain

**Keywords:** Orphan drugs, Pricing, Reimbursement, Spain

## Abstract

**Background:**

Pricing and reimbursement decisions for orphan drugs are faced with differences access between European countries depending on each reimbursement policies, evaluation processes and timings. In 2013, the therapeutic positioning report was introduced in the pricing and reimbursement process in Spain. The present study aims to identify orphan drugs authorised in Spain and approved by the European Commission between January 2003 and December 2019, analyse the impact of the therapeutic positioning report in the pricing and reimbursement process of orphan drugs in Spain and to assess additional potential criteria that could influence pricing and reimbursement decisions for orphan drugs.

**Results:**

Ninety-four orphan drugs have been approved by the European Commission between January 2003 and December 2019 and have marketing authorisation in Spain. Out of the 94 orphan drugs, 46 (48.9%) had received pricing and reimbursement approval. Before the inclusion of the therapeutic positioning report in year 2013, the mean time from European Commission approval to pricing and reimbursement approval for orphan drugs in Spain was 25.1 ± 16.5. After 2013, timelines have been reduced by an average of 9 months. The mean regulatory time from European Commission approval to Spanish marketing authorisation has decreased nearly 4 months (from 7.5 ± 10.2 months in years 2003–2013 to 3.8 ± 7.6 months in years 2014–2019). The instauration of the therapeutic positioning report could be associated with a reduction of the mean time from the Spanish marketing authorisation to pricing and reimbursement approval by an average of 5 months (from 17.3 ± 13.1 months in years 2003–2013 to 12.3 ± 5 months in years 2014–2019). In addition, orphan drugs with a positive conclusion in the therapeutic positioning report would be more likely to be reimbursed in Spain (*p* < 0,0001).

**Conclusions:**

This study shows that the therapeutic positioning report plays a key role in the pricing and reimbursement process in Spain. A positive conclusion of the therapeutic positioning report seems to favourably affect pricing and reimbursement decisions in Spain and, since its introduction, has also contributed to reduce pricing and reimbursement approval timelines in Spain.

## Background

Health Technology Assessment (HTA) country appraisals of Orphan Drugs (ODs) face added challenges due to the intrinsic characteristics of rare diseases from a clinical and economic point of view. The limited knowledge and heterogeneity of the diseases, the limitations in clinical trial development due to small and typically heterogeneous patient populations and the use of indirect endpoints [1] make it difficult to demonstrate added clinical benefit [2, 3].

While orphan designation and marketing authorisation occurs at European level, access to ODs remains a member state responsibility [4], resulting in differences and restrictions in access for patients due to differences in national reimbursement policies, processes and timings [5, 6].

Rare Diseases are a concerning health problem in Spain as they affect altogether about 3 million of patients (6,5% of the Spanish population) [7]. Reimbursement criteria for innovative medicines (including ODs) are explicitly defined in the Spanish legislation [8]. However, application in practice still remains unclear despite recent steps taken by the Spanish Ministry of Health (MoH) towards increased transparency in Pricing and Reimbursement (P&R) decisions: the Interministerial Committee on Pricing of Medicines and Healthcare Products (“CIPM” from Spanish initials), the ultimate P&R decision-maker, publishes a short summary of decisions to justify P&R approval or refusal based on current legislation [9].

In May 2013, a major change was introduced in the P&R process in Spain: The Therapeutic Positioning Report (TPR). The TPR is an evaluation document issued by the Spanish Agency of Medicines and Medical Devices (“AEMPS” from Spanish initials) that aims at determining the adequate positioning relative to what constitutes standard of care for the same indication to inform P&R decisions in Spain. The TPR includes a thorough review and summary of relative efficacy and safety data available for the new product. The Therapeutic Positioning Coordination Group (“GCPT” from Spanish initials), comprised by the AEMPS, the Directorate-General for the Basic Portfolio of Services of the National Healthcare and Pharmacy System (“DGCBF” from Spanish initials) and the Directorate-General for the Basic Portfolio of Services of the Autonomous Communities (CCAA), coordinates the TPR elaboration as soon as a new drug obtains a positive opinion from the Committee for Medicinal Products for Human Use (CHMP) and begins to work on a draft as soon as the Marketing Authorisation Holder (MAH) ask for P&R in Spain. This draft is confidential and is shared with the drug’s manufacturer and with relevant scientific societies and patient associations so that they can contribute with comments during a one-time allegations phase with a 10 days duration. One the final TPR draft is generated, it is sent to the DGCBF to inform P&R decision-making. Official TPR elaboration timelines estimate 3 months for the TPR to be drafted. In practice, and based on current metrics, the process can take up to 5 months. The final version of the TPR is published on the AEMPS website only after the P&R process has been completed and it includes the final P&R decision. The TPR initiative has contributed in making the P&R process more transparent but its real impact on the process has not been assessed and remains unclear.

As a result from previous phases of this study, we analysed potential criteria that could drive P&R decisions for ODs in Spain by analysing ODs approved by the European Commission (EC) between January 2012 and June 2018 that had marketing authorisation in Spain [10]. A total of 64 ODs were included in the analysis, from which only 28 (44.4%) were reimbursed in Spain and the rest were either undergoing a lengthy decision process or had been rejected. Authors found that a positive TPR conclusion and the existence of no therapeutic alternatives for the evaluated drug were drivers for P&R approval in Spain, implying that the TPR had become an important step in the Spanish P&R process.

This study aims to identify ODs authorized in Spain and approved by the EC between January 2003 and December 2019, analyse the impact of the TPR in the P&R process of ODs in Spain and to assess additional potential criteria that could influence P&R decisions for ODs.

## Results

### Identification of orphan drugs authorised in Spain and approved by the European Commission between 2003 & 2019 and description of their pricing & reimbursement situation in Spain

A total of 103 ODs approved by the EC between January 2003 and December 2019 were identified, of which 94 (91.3%) had been granted marketing authorization in Spain.

Out of the 94 ODs that were authorised in Spain, 46 (48.9%) had received P&R approval, 19 (20.2%) were undergoing the P&R process and 29 (30.9%) had their P&R request rejected. Of these, only 8 ODs were commercialised in the private market: Alprolix® [11], Bronchitol® [12], Holoclar® [13], Idelvion® [14], NexoBrid® [15], Procysbi® [16], Tobi Podhaler® [17] and Xermelo® [18].

The mean time from EC approval to P&R approval for ODs in Spain was 20.4 ± 13.1 months, with a minimum of 4 months (Kymriah® [19]) and a maximum of 61 months (Revestive® [20]). Before the inclusion of the TPR in year 2013, the mean time from EC approval to P&R approval for ODs in Spain was 25.1 ± 16.5. After the inclusion of the TPR during P&R process in Spain in 2013, timelines have been reduced by an average of 9 months.

**Fig. 1:**
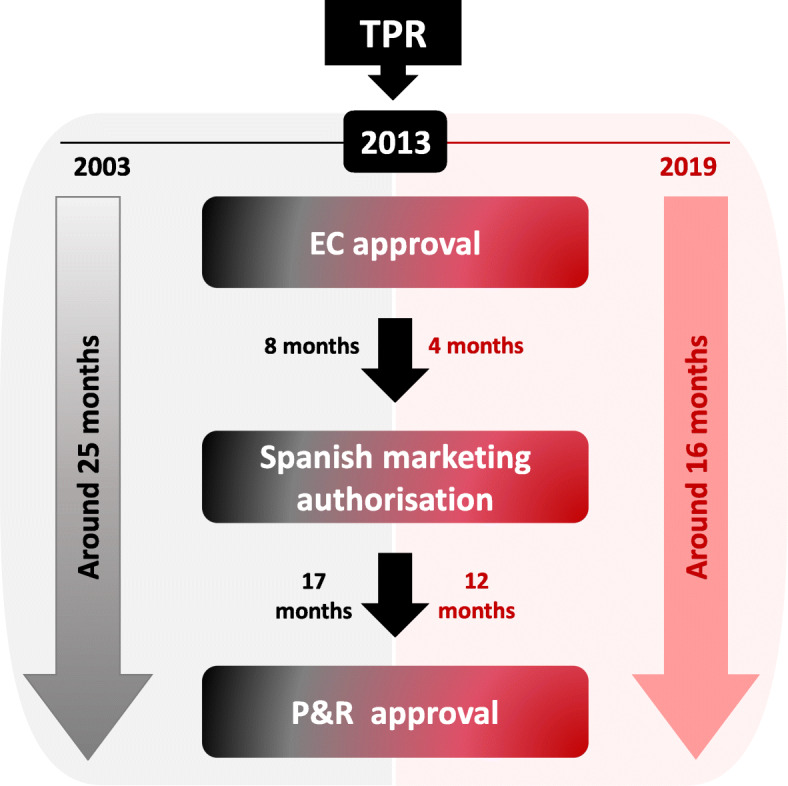
P&R timelines have been reduced after the inclusion of the TPR. The mean regulatory and P&R times of approved EC ODs from 2003 to 2019 from EC approval to P&R approval in Spain, stratified by before (*n* = 20) or after (*n* = 26) the inclusion of the TPR during P&R process in Spain in 2013

After 2013, the mean regulatory time from EC approval to Spanish marketing authorisation has decreased by an average of 4 months (from 7.5 ± 10.2 months in years 2003–2013 to 3.8 ± 7.6 months in years 2014–2019). The inclusion of TPRs during P&R negotiations in Spain has reduced the mean time from the Spanish marketing authorisation to P&R approval by an average of 5 months (from 17.3 ± 13.1 months in years 2003–2013 to 12.3 ± 5 months in years 2014–2019) (Fig. 1).

### Clinical and regulatory variables relevant for the pricing & reimbursement process in Spain

Results from identification of clinical and regulatory variables along with the reimbursement status of each OD are shown in Table 1. From the total of 94 studied ODs authorised in Spain for treating rare diseases, 34 ODs (36.2%) were indicated for oncologic diseases, 62 ODs (66%) had already therapeutic alternative indicated for treating the same condition, 57 ODs (60.6%) were indicated for rare diseases with a prevalence of < 5/10,000 inhabitants, 38 ODs (40.4%) had hard clinical trial outcomes, 53 ODs (56.4%) had a superior efficacy profile, 74 ODs (78.7%) did not have the obligation by the European Medicines Agency (EMA) to conduct a Post-authorisation safety study (PASS) and 53 ODs (56.4%) were indicated for adult patients.

**Table 1:** Identified variables for each orphan drug authorised in Spain and approved by the European Commission between 2003 & 2019

Brand name	P&R status in Spain	Clinical variables							Regulatory variables	
		Therapeutic Area	Existence of therapeutic alternatives	Rarity of disease	Outcomes classification	Efficacy profile	Safety profile*	Type of population	TPR conclusion	Conditional approval
Adcetris®	Approved	Oncologic	Yes	Ultra-rare	Hard	Similar	No	Adults	Positive	Yes
Adempas®	Approved	Other	No	Ultra-rare	Medium	Superior	No	Adults	Positive	No
Alofisel®	Approved	Oncologic	Yes	Rare	Soft	Similar	No	Adults	Positive	No
Alprolix®	Rejected	Other	Yes	Ultra-rare	Medium	Similar	No	All ages	Positive	No
Besponsa®	Approved	Oncologic	No	Rare	Hard	Superior	No	Adults	Positive	No
Blincyto®	Rejected	Oncologic	Yes	Rare	Hard	Similar	Yes	All ages	Positive	No
Brineura®	Under P&R decision process	Other	No	Ultra-rare	Medium	Similar	Yes	All ages	Not published	No
Bronchitol®	Rejected	Other	Yes	Rare	Medium	Similar	No	All ages	Not published	No
Cablivi®	Under P&R decision process	Other	Yes	Ultra-rare	Soft	Superior	No	Adults	Not published	No
Carbaglu®	Approved	Other	Yes	Ultra-rare	Soft	Superior	Yes	Adults	Not published	No
Cerdelga®	Approved	Other	Yes	Ultra-rare	Soft	Superior	Yes	Adults	Positive	No
Chenodeoxycholic acid Leadiant®	Under P&R decision process	Other	No	Ultra-rare	Soft	Similar	No	All ages	Not published	No
Cometriq®	Rejected	Oncologic	Yes	Ultra-rare	Hard	Similar	No	Adults	Positive	Yes
Cresemba®	Approved	Other	Yes	Ultra-rare	Hard	Similar	No	Adults	Positive	No
Crysvita®	Under P&R decision process	Other	No	Ultra-rare	Medium	Similar	Yes	Paediatric	Not published	Yes
Cystadrops®	Rejected	Other	Yes	Ultra-rare	Soft	Superior	No	All ages	Positive	No
Dacogen®	Approved	Oncologic	Yes	Rare	Hard	Superior	No	Adults	Positive	No
Darzalex®	Approved	Oncologic	Yes	Rare	Hard	Superior	No	Adults	Positive	No
Deltyba®	Approved	Other	Yes	Rare	Soft	Similar	No	Adults	Positive	Yes
Epidyolex®	Under P&R decision process	Other	Yes	Ultra-rare	Hard	Similar	No	All ages	Not published	No
Esbriet®	Approved	Oncologic	No	Rare	Medium	Similar	No	Adults	Positive	No
Farydak®	Rejected	Oncologic	Yes	Rare	Hard	Similar	No	Adults	Negative	No
Firazyr®	Approved	Other	Yes	Rare	Medium	Similar	No	All ages	Not published	No
Firdapse®	Rejected	Other	No	Ultra-rare	Hard	Superior	No	Adults	Not published	No
Galafold®	Approved	Other	Yes	Ultra-rare	Soft	Similar	No	Adults	Positive	No
Gazyvaro®	Approved	Oncologic	Yes	Rare	Hard	Superior	No	Adults	Positive	No
Granupas®	Rejected	Other	No	Rare	Medium	Similar	No	All ages	Not published	No
Holoclar®	Rejected	Other	No	Rare	Hard	Similar	No	All ages	Negative	Yes
Iclusig®	Approved	Oncologic	No	Rare	Soft	Similar	Yes	Adults	Not published	No
Idelvion®	Rejected	Other	Yes	Ultra-rare	Hard	Similar	No	All ages	Positive	No
Imbruvica®	Approved	Oncologic	Yes	Rare	Hard	Similar	No	Adults	Positive	No
Imnovid®	Approved	Oncologic	Yes	Rare	Hard	Superior	Yes	Adults	Positive	No
Jorveza®	Rejected	Other	Yes	Rare	Soft	Superior	No	Adults	Negative	No
Kalydeco®	Approved	Other	No	Rare	Medium	Similar	No	All ages	Positive	No
Kanuma®	Approved	Other	No	Ultra-rare	Hard	Superior	Yes	All ages	Positive	No
Kuvan®	Approved	Other	Yes	Rare	Soft	Superior	No	All ages	Not published	No
Kymriah®	Approved	Oncologic	Yes	Rare	Hard	Similar	Yes	All ages	Positive	No
Kyprolis®	Approved	Oncologic	Yes	Rare	Hard	Superior	No	Adults	Positive	No
Lamzede®	Under P&R decision process	Other	No	Ultra-rare	Soft	Superior	No	All ages	Not published	No
Ledaga®	Rejected	Oncologic	Yes	Rare	Hard	Superior	No	Adults	Negative	No
Lutathera®	Approved	Other	Yes	Ultra-rare	Hard	Superior	No	Adults	Positive	No
Luxturna®	Under P&R decision process	Other	No	Rare	Medium	Superior	Yes	All ages	Not published	No
Mepsevii®	Under P&R decision process	Other	No	Ultra-rare	Soft	Similar	No	All ages	Not published	No
Mozobil®	Approved	Oncologic	Yes	Rare	Medium	Superior	No	All ages	Not published	No
Myalepta®	Rejected	Other	Yes	Ultra-rare	Soft	Similar	No	All ages	Not published	No
Mylotarg®	Approved	Oncologic	No	Rare	Hard	Superior	No	All ages	Positive	No
Namuscla®	Under P&R decision process	Other	No	Rare	Hard	Superior	No	Adults	Not published	No
Natpar®	Rejected	Other	Yes	Ultra-rare	Soft	Superior	No	Adults	Negative	Yes
Nexavar®	Approved	Oncologic	Yes	Rare	Hard	Superior	No	All ages	Not published	No
NexoBrid®	Rejected	Other	No	Rare	Medium	Superior	Yes	Adults	Not published	No
Ninlaro®	Rejected	Oncologic	Yes	Rare	Hard	Superior	No	Adults	Negative	Yes
Ocaliva®	Approved	Other	Yes	Ultra-rare	Soft	Superior	No	Adults	Positive	Yes
Ofev®	Approved	Oncologic	No	Rare	Medium	Similar	No	Adults	Positive	No
Onivyde®	Approved	Oncologic	Yes	Rare	Hard	Superior	No	Adults	Positive	No
Onpattro®	Under P&R decision process	Other	Yes	Rare	Hard	Superior	No	Adults	Not published	No
Opsumit®	Approved	Other	Yes	Ultra-rare	Hard	Superior	No	Adults	Positive	No
Orphacol®	Approved	Other	No	Ultra-rare	Hard	Similar	No	All ages	Not published	No
Oxervate®	Rejected	Other	No	Rare	Hard	Superior	No	Adults	Negative	No
Palynziq®	Under P&R decision process	Other	Yes	Rare	Soft	Superior	Yes	All ages	Not published	No
Plenadren®	Rejected	Other	Yes	Rare	Soft	Similar	No	Adults	Not published	No
Poteligeo®	Under P&R decision process	Oncologic	Yes	Rare	Hard	Superior	No	Adults	Not published	No
Prevymis®	Rejected	Other	Yes	Rare	Medium	Superior	No	Adults	Negative	No
Procysbi®	Rejected	Other	Yes	Ultra-rare	Soft	Similar	No	All ages	Negative	No
Qarziba®	Under P&R decision process	Oncologic	Yes	Rare	Soft	Similar	Yes	Paediatric	Not published	No
Ravicti®	Approved	Other	Yes	Rare	Soft	Similar	Yes	All ages	Not published	No
Raxone®	Under P&R decision process	Other	No	Rare	Medium	Similar	No	All ages	Not published	No
Revestive®	Approved	Other	No	Ultra-rare	Medium	Superior	Yes	Adults	Not published	No
Rydapt®	Approved	Oncologic	Yes	Ultra-rare	Hard	Superior	No	Adults	Positive	No
Scenesse®	Rejected	Other	No	Ultra-rare	Soft	Superior	No	Adults	Not published	No
Signifor®	Approved	Other	Yes	Rare	Soft	Superior	No	Adults	Positive	No
Sirturo®	Rejected	Other	Yes	Rare	Soft	Superior	No	Adults	Positive	Yes
Soliris®	Approved	Oncologic	Yes	Rare	Medium	Superior	No	All ages	Positive	No
SomaKit TOC®	Approved	Other	Yes	Rare	Soft	Similar	No	Adults	Positive	No
Spinraza®	Approved	Other	No	Ultra-rare	Hard	Superior	No	All ages	Positive	No
Strensiq®	Rejected	Other	No	Ultra-rare	Soft	Similar	No	Paediatric	Positive	No
Sylvant®	Approved	Oncologic	No	Rare	Medium	Superior	Yes	Adults	Positive	No
Symkevi®	Approved	Other	Yes	Rare	Medium	Superior	No	All ages	Positive	No
Takhzyro®	Under P&R decision process	Other	Yes	Ultra-rare	Medium	Superior	No	All ages	Not published	No
Tegsedi®	Under P&R decision process	Other	Yes	Rare	Medium	Superior	No	Adults	Not published	No
Tepadina®	Approved	Oncologic	Yes	Ultra-rare	Hard	Similar	No	All ages	Not published	No
Tobi Podhaler®	Rejected	Other	Yes	Rare	Medium	Superior	No	All ages	Not published	No
Translarna®	Rejected	Other	No	Rare	Medium	Similar	No	All ages	Negative	Yes
Verkazia®	Under P&R decision process	Other	Yes	Rare	Soft	Superior	No	Paediatric	Not published	No
Vimizim®	Rejected	Other	No	Ultra-rare	Medium	Superior	Yes	All ages	Negative	No
Votubia®	Approved	Oncologic	No	Rare	Medium	Superior	No	Adults	Not published	No
Vpriv®	Approved	Other	Yes	Ultra-rare	Soft	Similar	No	All ages	Not published	No
Vyndaqel®	Approved	Other	No	Ultra-rare	Medium	Similar	Yes	Adults	Positive	No
Vyxeos®	Rejected	Oncologic	Yes	Rare	Hard	Superior	No	Adults	Not published	No
Wakix®	Rejected	Other	Yes	Rare	Hard	Similar	Yes	Adults	Positive	No
Xaluprine®	Under P&R decision process	Oncologic	Yes	Rare	Soft	Similar	No	All ages	Not published	No
Xermelo®	Rejected	Other	Yes	Rare	Soft	Superior	No	Adults	Not published	No
Xospata®	Under P&R decision process	Oncologic	No	Rare	Hard	Superior	No	Adults	Not published	No
Yescarta®	Approved	Oncologic	Yes	Rare	Hard	Similar	Yes	Adults	Positive	No
Zejula®	Approved	Oncologic	Yes	Ultra-rare	Hard	Superior	No	Adults	Positive	No

*P&R* Pricing and reimbursement; *TPR* Therapeutic positioning report. *Obligation or not to conduct a post-authorisation safety study (PASS)

**Fig. 2:**
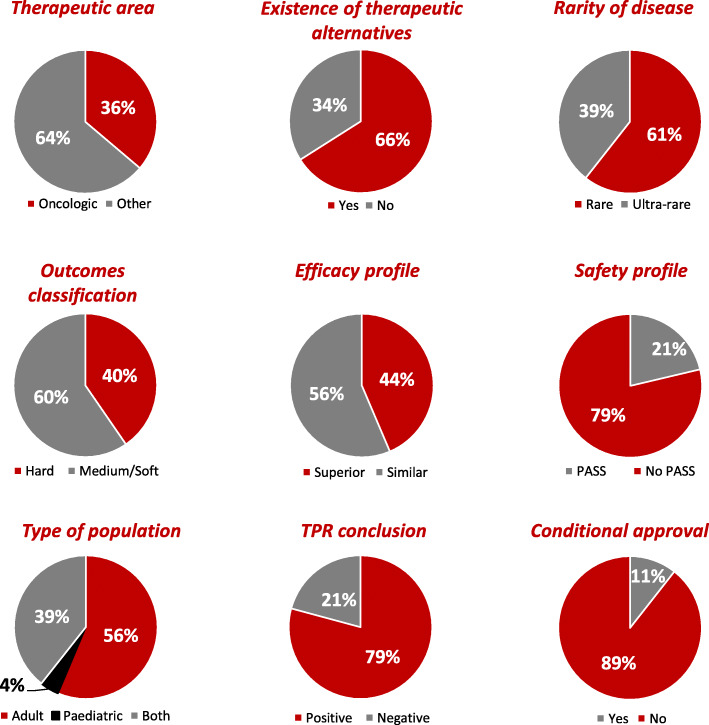
Results from identification of clinical and regulatory variables. Descriptive results of clinical and regulatory variables of ODs authorised in Spain for treating rare diseases and approved by the European Commission between 2003 & 2019 (*n* = 94). PASS, obligation by the EMA to conduct a Post-authorisation safety study; No PASS, no obligation by the EMA to conduct a Post-authorisation safety study

Out of the 53 published TPRs, 42 ODs (79.2%) had a positive conclusion. From the 94 studied ODs, 84 ODs (89.4%) were not granted conditional approval marketing authorisation by the EMA (Fig. 2).

### Clinical and regulatory variables relevant for the pricing & reimbursement process in Spain according to its reimbursement status

#### ODs for which P&R had been approved

Out of the 46 reimbursed ODs, 24 ODs (52.2%) were indicated for oncologic diseases, 32 ODs (69.6%) had a therapeutic alternative, 28 ODs (60.9%) were indicated for rare diseases with a prevalence of < 5/10,000 inhabitants, 22 ODs (47.8%) had hard clinical trial outcomes, 27 ODs (58.7%) had a superior efficacy profile, 35 ODs (76.1%) did not have the obligation by the EMA to conduct a PASS and 31 ODs (67.4%) were indicated for adult patients.

Out of the 34 ODs with a published TPR, 34 ODs (100%) had a positive TPR conclusion. Out of the 46 reimbursed ODs, 43 ODs (93.5%) were not granted conditional marketing authorisation by the EMA.

#### ODs for which P&R had been rejected

Out of the 29 P&R rejected ODs, 6 ODs (20.7%) were indicated for oncologic diseases, 20 ODs (69%) had a therapeutic alternative, 18 ODs (62.1%) were indicated for rare diseases with a prevalence of < 5/10,000 inhabitants, 11 ODs (37.9%) had hard clinical trial outcomes, 15 ODs (51.7%) had a superior efficacy profile, 25 ODs (86.2%) did not have the obligation by the EMA to conduct a PASS and 16 ODs (55.2%) were indicated for adult patients.

Out of the 19 ODs (65.5%) with a published TPR, 8 ODs (42%) had a positive TPR conclusion. Out of the 29 ODs without reimbursement, 23 ODs (79.3%) were not granted conditional marketing authorisation by the EMA.

### Analysis of potential relationship between clinical and regulatory variables and reimbursement status of ODs in Spain

The objective of the analysis was to test the validity of hypotheses described in the methodology section, and to identify variables that may positively influence reimbursement in Spain. A logistic regression model was used to predict the impact of the studied variables on reimbursement: existence of a therapeutic alternative, outcomes classification, efficacy profile, safety profile, TPR conclusion and conditional approval. The analysis included P&R approved and P&R rejected ODs in Spain (*n* = 75).

The TPR conclusion variable was clearly correlated to the reimbursement status for ODs in Spain (*p*-value < 0,0001). As a result, ODs with a positive TPR conclusion (ODs recommended for a group of patients or considered equivalent to an alternative approved in Spain) would be more likely to be reimbursed in Spain. The regression analysis results did not include the TPR conclusion variable due to perfect separation. The results of the univariate and multivariate regression model did not show a significant correlation between the studied variables and reimbursement status (Table 2).

**Table 2:** Results of the regression analysis

Variable	Estimate	95% CI	|Z|	***P*** value	***P*** value classification
**Univariate regression analysis**					
Existence of therapeutic alternative	1,029	0,3674 to 2,798	0,05485	0,9563	ns
Outcomes classification	1,5	0,5863 to 3,943	0,8389	0,4015	ns
Efficacy profile	0,754	0,2938 to 1,927	0,5917	0,554	ns
Safety profile	1,964	0,5944 to 7,735	1,055	0,2914	ns
Conditional approval	0,2674	0,0525 to 1,111	1,752	0,0798	ns
**Multivariate regression analysis**					
Existence of therapeutic alternative	1,129	0,3821 to 3,280	0,2231	0,8234	ns
Outcomes classification	1,518	0,57 to 4,165	0,8289	0,4072	ns
Efficacy profile	0,8212	0,3092 to 2,193	0,3968	0,6915	ns
Safety profile	1,731	0,4964 to 7,141	0,8238	0,41	ns
Conditional approval	0,2995	0,057 to 1,292	1,562	0,1182	ns

The dependent variable in logistic regression was reimbursement status, stratified by P&R approved or P&R rejected (*n* = 75). *CI* Confidence interval; *Z* Z-score; ns: not significant

The variable that has the largest impact on the probability of reimbursement in Spain is the TPR conclusion. This means that ODs with a positive TPR conclusion are more likely to be reimbursed.

## Discussion

A total of 94 ODs have been approved by the EC between January 2003 and December 2019 and have marketing authorisation in Spain. The mean time from EC approval to P&R approval for ODs in Spain was 20.4 ± 13.1 months and the mean time from Spanish marketing authorisation to P&R approval was 14 ± 9.74 months. Based on the results of the study, having the EC and Spanish marketing authorisation approval does not guarantee access within the Spanish market, as from the 94 studied ODs, 46 (48.9%) were reimbursed in Spain at the moment of the study, and the rest of ODs were either undergoing the decision process or rejected, which prevents patients equitable and timely access to these drugs.

This study shows that the only studied variable that seems to affect P&R decisions in Spain is the conclusion of the TPR. This variable has been the only are consistently showing statistical significance across different analyses of the present study considering different time periods [10], demonstrating the key role that the TPR plays in the P&R process. It is important to highlight that the TPR, since its introduction in 2013, has also contributed to reduce P&R approval timelines in Spain by an average of 9 months. After 2013, the mean regulatory time from EC approval to Spanish marketing authorisation has decreased nearly 4 months, indicating that the MAH could be asking for P&R in Spain sooner than in the past and the administrative process might have been accelerated. Authors do not think that the reduction in regulatory timelines from EC approval to Spanish marketing authorisation is related to the introduction of the TPR. Additionally, the introduction of the TPR during P&R negotiations in Spain could be associated with a reduction of the mean time from the Spanish marketing authorisation to P&R approval by an average of 5 months.

A recent study that assessed the access to orphan medicines in Spain until September 2019 [21] has reported similar findings to the present study in terms of the identified OD sample, their P&R status and estimated regulatory times during the P&R process, thus reinforcing the validity of the data presented in this study. However, the above-mentioned study did not assess the potential influence of different variables in P&R decisions.

None of clinical variables related to P&R criteria set in the Spanish legislation have been found to directly affect P&R decisions. This reinforces the widely held opinion in the published literature highlighting the lack of transparency and availability of information with regards to which criteria are used in real life for P&R evaluation and decision-making of ODs across European countries [4, 6].

In recent years, actions have been made at international level to try to reduce uncertainty surrounding the appraisal of ODs and to increase the process’ transparency, like the creation of specific frameworks to assess ODs [22] or the publication of recommendations on principles to help improve the consistency of ODs P&R assessment in Europe [23]. A recent publication from Paulden et al. [22] identified decision criteria that could influence P&R of ODs, such as the availability of therapeutic alternatives, the evidence of clinical efficacy, the severity of the disease or the impact of treatment on life expectancy and quality of life. Another highlighted point by Paulden et al. [22] is the diversity of views around P&R decision criteria, depending on the context. Therefore, it would be important and beneficial to incorporate preferences from several stakeholders when making P&R decisions.

The recent creation of specific frameworks for OD appraisal, including the recent creation of a framework for the evaluation of ODs in Spain at national level [8, 24] using Multiple-Criteria Decision Analysis (MCDA) methodology [25], could contribute to ensuring a systematic and transparent evaluation process for ODs’ P&R, aligned with the criteria set in the Spanish legislation, and incorporate preferences from several stakeholders when making P&R decisions.

Although there is still a long way to go towards total transparency in the P&R process in Europe and Spain, it should be noted that important advances have been made in recent years that have shed some light and contributing to optimise the P&R process for innovative medicines in Spain. Important examples include the introduction of the TPR, which incorporates different stakeholder perspectives and has reduced the average time of the P&R process and has made the Spanish MoH clinical positioning on the evaluated drug transparent and publicly available. Another important achievement is the publication of the CIPM agreements, where the favourable and unfavourable P&R decisions are related to the criteria contemplated in the Spanish legislation justifying the favourable or non-favourable decision. Also, the BIFIMED database launched in 2019 [26], which publishes all information regarding the reimbursement of a medicine (except its price), thanks to which we have been able to improve the quality of the data related to P&R approval times with respect to our previous study [10], where we had to collect this information using indirect sources.

### Study limitations

The studied variables might not take into account all the P&R criteria that Spanish evaluators take into consideration, such as severity of the disease, unmet needs of specific populations, therapeutic and social drug value, incremental clinical benefit taking into account cost-effectiveness, budget impact, existence of alternative treatment options for the indication and degree of innovation, because of the difficulty in comparing and classifying using uniform criteria all the different types of ODs and rare diseases.

Important economic variables that could affect P&R decisions, like drug price and budget impact (BI) could not be assessed due to the lack of validity of the information available regarding real-life reimbursement. First, the P&R process is not fully transparent in Spain and the sales forecasts that the manufacturers send to the Ministry of Health are not publicly available. Second, the exact number of patients eligible for each OD’s indication was not known. Prevalence data for the majority of rare diseases is not known and, in many cases, there is no published prevalence for the OD’s exact indication.

There was not enough power in the study for further analysis. A future analysis of clinical and regulatory variables involving a larger sample size of ODs authorised in Spain is needed to further explore their impact on the P&R process.

## Conclusions

From all ODs approved by the EC and which had obtained Marketing Authorisation in Spain, 46 (48.9%) were reimbursed, 19 (20.2%) were undergoing decision and 29 (30.9%) were rejected. The Spanish regulatory timelines for ODs have been reduced after the inclusion of the TPR during P&R process by an average of 9 months. The mean regulatory time from EC approval to Spanish marketing authorisation has decreased by an average of 4 months and the mean time from the Spanish marketing authorisation to P&R approval has decreased by an average of 5 months. A positive TPR conclusion is a key driver for P&R approval for ODs. In addition, oncology ODs might be more likely to be reimbursed in Spain. Economic variables such as the price of the drug and the total budget impact derived from its introduction were not assessed in this study because of lack of transparency and lack of validity of publicly available information. Official listed prices in the available databases do not reflect the reimbursement price agreed between the Ministry of health and the MAH.

## Methods

A protocol, including an analysis plan, was developed before the development of the present study, which includes the study’s hypothesis, variables (and stratification of variables) and pre-specified statistical analyses.

All collected data and statistical analyses were included in an internal data base.

### Identification of European Commission approved orphan drugs between 2003 & 2019

ODs to treat rare diseases with current orphan designations were retrieved from the European Community Register of orphan medicinal products [27]. ODs with orphan designations that have been expired or removed by the sponsor were excluded from the study. EC approved ODs and their EC approval dates were extracted from the EMA’s website [28] through their online medicine finder engine, with the following search filters: human medicines, orphan medicines and authorized medicines. The ODs found were grouped according to the EC authorization year 2003 to 2019.

### Identification of orphan drugs authorised in Spain and their pricing & reimbursement situation

ODs authorised in Spain were retrieved from the Spanish Medicine Online Information Centre (CIMA) of the AEMPS [29]. This study only included drugs that had been granted marketing authorization in Spain. The CIMA database was used to search if the OD had a Spanish marketing authorisation, its authorisation date and commercialisation status in Spain. The Spanish marketing authorisation dates were used to analyse the time from EC approval to Spanish marketing authorisation and the time from Spanish marketing authorisation to P&R approval date.

The BIFIMED database was used to search each OD P&R status information [26]. ODs were classified into three reimbursement categories: P&R approved (ODs that have had their P&R request approved), under P&R decision process (ODs that have requested P&R but are still under P&R negotiations) and P&R rejected (ODs that have seen their P&R request rejected). P&R approval dates were used to analyse the time from the Spanish marketing authorisation to P&R approval in Spain.

### Identification and description of relevant variables for the pricing & reimbursement process in Spain

#### Clinical variables

Clinical variables were identified based on formal and informal criteria used in the Spanish P&R process [30–33], which were tested in previous phases of this study [10]. The studied clinical variables are part of the mandatory clinical information that the MAH must provide to the Spanish P&R regulation bodies: (I) Therapeutic area (ODs were divided into two groups, oncology or other, according to their indication and taking in consideration the Anatomical Therapeutic Chemical (ATC) code), (II) Existence of therapeutic alternatives (ODs were classified into two groups depending on whether a therapeutic alternative was available, known as drugs indicated for treating the same condition), (III) Rarity of disease (Indication’s prevalence was analysed and categorised into rare diseases, that affect < 5/10,000 inhabitants, or ultra-rare diseases, that affect < 1/50,000 inhabitants. Spanish prevalence data were used when available), (IV) Outcomes classification (The classification of clinician-reported outcomes assessment (COAs) proposed by Powers III, JH. et al. was used [34]. ODs clinical trial outcomes were analysed and classified into hard (measures of survival and patient reported outcomes, PRO), intermedium (functional capacity tests and other clinical reported outcomes, CRO) or soft (outcomes assessments using biomarkers)), (V) Efficacy profile (ODs clinical trials were analysed and classified into similar (trial uncontrolled or statistically significantly non-superior efficacy compared with placebo and non-superior efficacy compared with active comparator) or superior (statistically significantly superior efficacy compared with placebo and statistically significantly superior efficacy compared with active comparator) efficacy profile), (VI) Safety profile (ODs were classified into two groups depending on whether they had the obligation by the EMA to conduct a Post-authorisation safety study, PASS) and (VII) Type of population (Categorised into paediatric, adults or both).

Hypothesis were defined for the following clinical variables: (I) Existence of therapeutic alternatives (ODs indicated for a disease without any therapeutic alternative would be more likely to have P&R approval, as these patients present high unmet clinical needs compared to patients that can be currently treated), (II) Outcomes classification (ODs with hard outcomes would be more likely to have P&R approval than drugs with intermedium or soft outcomes since they will represent less uncertainty for evaluators and decision makers), (III) Efficacy profile (ODs that showed a superior efficacy profile would be more likely to be reimbursed) and (IV) Safety profile (ODs that showed a safety profile that raised less uncertainty among evaluators would be more likely to be reimbursed).

The clinical variables (therapeutic area, existence of therapeutic alternatives and rarity of disease) were extracted from the corresponding TPR [35] and/ or the European public assessment report (EPAR) [28]. When no information was available on the prevalence of the diseases, a search in biomedical databases and/ or grey literature was performed and prevalence data was extracted from published epidemiology studies. Outcomes classification, efficacy profile, safety profile and type of population were extracted from the clinical trials mentioned in the EPAR [28].

#### Regulatory variables

Regulatory variables were identified, based on formal and informal criteria used in the Spanish P&R process [30–33] which were tested in previous phases of this study [10]. The studied regulatory variables are part of the regulatory process in Europe or Spain: (I) TPR conclusion (ODs TPR conclusions were analysed and then categorised into positive (ODs recommended to a group of patients or equivalent to an alternative approved in Spain), negative conclusion (ODs not recommended) or missing data (ODs authorized before May 2013 or with their TPR in process)) and (II) Conditional approval (ODs may be granted a conditional marketing authorisation by the EMA. ODs were classified into two groups depending on whether they had a conditional marketing authorisation or not).

Hypothesis were defined for the following regulatory variables: (I) TPR conclusion (ODs with a published TPR with a positive conclusion would be more likely to be reimbursed in Spain) and (VI) Conditional approval (ODs with a conditional approval by the EMA would be less likely to be reimbursed in Spain).

The AEMPS’s webpage was used to search information about the publication of the TPR for each OD [35]. The AEMPS started elaborating TPRs in 2013, hence, not all ODs in this study had a TPR available driving the P&R decision process. The EMA’s website [28] was used to search the conditional approval status for each OD.

### Statistical analysis

A descriptive analysis of the clinical and regulatory variables was conducted and further stratified by P&R status including ODs authorised in Spain and approved by the EC between 2003 and 2019. Quantitative data (including time from EC approval to Spanish marketing authorisation, from Spanish marketing authorisation to P&R approval and time from Spanish marketing authorisation to P&R decision process to 31/12/2019) were described through basic statistic descriptive analysis. Qualitative data were described and stratified by P&R status.

In order to answer our hypothesis, a statistical analysis of the clinical and regulatory variables was conducted. The objective of the analysis was to test the validity of the previous hypothesis and to identify variables that may positively influence the reimbursement in Spain. A logistic regression model [36] was used to predict the impact of the studied variables on reimbursement in Spain: existence of therapeutic alternatives, outcomes classification, efficacy profile, safety profile, TPR conclusion and conditional approval. Each variable was analysed using an univariate regression model. Additionally, variables were analysed using a multivariate regression model. The analysis included P&R reimbursed and P&R rejected ODs in Spain (*n* = 75).

The software used to conduct all statistical analysis was GraphPad Prism 8 (Version 8.3.0).

## Data Availability

In 2017, Omakase Consulting S.L. developed an OD database to collect data related to medicinal products with OD designation, currently authorised in Europe and their P&R situation in Spain. The datasets used and analysed during the current study are available from the corresponding author on reasonable request.

## Abbreviations

AEMPS: Spanish Agency of Medicines and Medical Devices
ATC: Anatomical Therapeutic Chemical
BI: Budget Impact
CCAA: Autonomous communities
CHMP: Committee for Medicinal Products for Human Use
CIMA: Spanish Medicine Online Information Centre
CIPM: Interministerial Committee on Pricing of Medicines and Healthcare Products
CRO: Clinical Reported Outcome
DGCBF: Directorate-General for the Basic Portfolio of Services of the National Health and Pharmacy System
EC: European Commission
EMA: European Medicines Agency
EPAR: European Public Assessment Report
GCPT: Therapeutic Positioning Coordination Group
HTA: Health Technology Assessment
MAH: Marketing Authorisation Holder
MCDA: Multiple-Criteria Decision Analysis
MoH: Ministry of Health
OD: Orphan Drug
P&R: Pricing and Reimbursement
PASS: Post-authorisation Safety Study
PRO: Patient Reported Outcome
TPR: Therapeutic Positioning Report
